# Changes in lymphocyte subsets in patients with Guillain-Barré syndrome treated with immunoglobulin

**DOI:** 10.1186/s12883-014-0202-3

**Published:** 2014-10-15

**Authors:** Hui Qing Hou, Jun Miao, Xue Dan Feng, Mei Han, Xiu Juan Song, Li Guo

**Affiliations:** Department of Neurology, the Second Hospital of Hebei Medical University, Key laboratory of Hebei Neurology, Shi jia zhuang, Hebei 050000 China; Department of Neurosurgery, the General Hospital of North China Petroleum Administration Bureau, Ren qiu, HeBei 062550 China; Emergency Department, the Second Hospital of Hebei Medical University, Shi jia zhuang, Hebei 050000 China

**Keywords:** Acute inflammatory demyelinating polyneuropathy, Acute motor axonal neuropathy, Guillain-Barré syndrome, Intravenous immunoglobulin, Lymphocyte subsets

## Abstract

**Background:**

Guillain-Barré syndrome (GBS) is an autoimmune condition characterized by peripheral neuropathy. The pathogenesis of GBS is not fully understood, and the mechanism of how intravenous immunoglobulin (IVIG) cures GBS is ambiguous. Herein, we investigated lymphocyte subsets in patients with two major subtypes of GBS (acute inflammatory demyelinating polyneuropathy, AIDP, and acute motor axonal neuropathy, AMAN) before and after treatment with IVIG, and explored the possible mechanism of IVIG action.

**Methods:**

Sixty-four patients with GBS were selected for our study and divided into two groups: AIDP (n = 38) and AMAN (n = 26). Thirty healthy individuals were chosen as the control group. Relative counts of peripheral blood T and B lymphocyte subsets were detected by flow cytometry analysis.

**Results:**

In the AIDP group, the percentage of CD4^+^CD45RO^+^ T cells was significantly higher, while the percentage of CD4^+^CD45RA^+^ T cells was notably lower, than in the control group. After treatment with IVIG, the ratio of CD4^+^/CD8^+^ T cells and the percentage of CD4^+^CD45RA^+^ T cells increased, while the percentages of CD8^+^ T cells and CD4^+^CD45RO^+^ T cells decreased significantly, along with the number of CD19^+^ B cells. However, there were not such obvious changes in the AMAN group. The Hughes scores were significantly lower in both the AIDP and AMAN groups following treatment with IVIG, but the changes in Hughes scores showed no significant difference between the two groups.

**Conclusions:**

This study suggested that the changes in T and B-lymphocyte subsets, especially in CD4^+^T-lymphocyte subsets, might play an important role in the pathogenesis of AIDP, and in the mechanism of IVIG action against AIDP.

## Background

Guillain-Barré syndrome (GBS) is an acute, immune-mediated attack on the peripheral nervous system that leads to flaccid paralysis, with a case fatality rate of 5–10% [1]. Both cellular and humoral immunity participate in the onset of GBS, though cellular immunity is the primary cause [2]. Based on clinical, electrophysiological, and pathologic characteristics, GBS can be divided into two major subtypes: AIDP (acute inflammatory demyelinating polyneuropathy, AIDP) and AMAN (acute motor axonal neuropathy, AMAN) [3]. At present there is no specific treatment for GBS; intravenous immunoglobulin (IVIG) has been the drug of choice for GBS treatment because it provides the most effective clinical results [4,5], with almost no contraindications.

Lymphocyte function is related to the numerous complex superficial cell membrane proteins on the cell surface. As a result, lymphocyte immune phenotype analysis can be used as an important reference index for evaluating the body’s immune status. In recent years, much work has been undertaken to study the distribution of lymphocyte subsets in GBS, but the results are variable [6-9]. Our team found that the distribution of lymphocyte subsets differs greatly between individuals; therefore, it is important to test individual GBS patients both before and after IVIG treatment [5]. In the current study, we examined GBS in a population of individuals from northern China who developed AIDP or AMAN. This study used a matched-pairs design using each patient’s own pre- and post-treatment data. We detected changes in T and B lymphocyte subset distribution, allowing us to explore the pathogenesis of GBS and speculate on the mechanism of IVIG in treating GBS.

## Methods

### Patients

All subjects (patients with AIDP or AMAN and healthy controls) were from northern China and were referred to the Second Hospital of Hebei Medical University in Shijiazhuang from 2010–2013. All patients fulfilled accepted diagnostic criteria [10], and were studied within 2 weeks of the onset of GBS. Sixty-four cases underwent electrophysiological examination, being recorded for motor conductive velocity (MCV), distal latency, F wave, and motor evoked amplitude [10-13], and were classified into two groups: AIDP (*n* =38) and AMAN (*n* =26). The primary outcome parameter was GBS disability (Hughes) scale score at discharge. Thirty subjects (age- and sex-matched controls) from the same area were also included in the study. Controls had no personal or family history of GBS, and no sign of any peripheral neuropathy. Controls were chosen randomly.

Peripheral blood was collected and T and B lymphocyte subset relative counts were detected by flow cytometry both before and after treatment with IVIG. This study protocol was approved by the Research Ethics Committee of the Second Hospital of Hebei Medical University and followed the ethical guidelines of the 1975 Declaration of Helsinki and all subsequent modifications [14]. All patients provided a written informed consent to participate in this research.

### Therapeutic method

Patients were treated with IVIG (0.4 g·kg^−1^·d^−1^) continuously for 5 days. At 3 weeks post-therapy, patients were again graded using the Hughes scale [15,16].

### Flow cytometry

Prior to therapy, and again within 24 hours of the final therapy with IVIG, whole blood was collected in EDTA vacutainer tubes. Cyflow reagents and consumables were used according to the manufacturer’s protocol. The set comprised the following antibodies: CD4-APC/CD8-PE/CD3-FITC; CD4-APC/CD45RA-FITC/CD45RO-PE; CD19-FITC (Becton Dickinson, San Jose, CA, USA). Meanwhile, IgG1-FITC/IgG2a-PE was replied as isotype control. 100 μl of the blood was incubated in tubes together with 20 μl of the antibodies. The incubation was performed in the dark, at room temperature for 15 min. After incubation, erythrocytes were subsequently lysed, and the cell suspension was centrifuged, washed three times, and resuspended in an appropriate volume of flow staining buffer. A minimum of 10,000 cells was accepted for FACS (BD Biosciences, San Jose, CA, USA) analysis. Cells were gated based on morphological characteristics.

### Analysis

Statistical analyses were conducted using SPSS18.0 software, and continuous variables are expressed as mean ± standard deviation ($\overline{x}\pm s$). The mean differences between the two samples were analyzed using a t-test. The mean differences between the two patient groups before and after treatment were compared using a paired t-test. The inspection level was *α* =0.05 and differences were considered significant at *p* <0.05.

## Results

**Figure 1:**
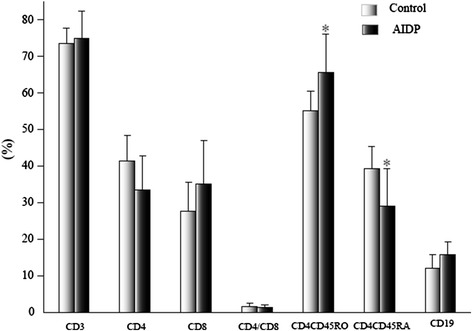
**Comparison of lymphocyte subsets in AIDP and control groups before treatment.** The data are mean ± S.D. *p <0.05, relative to the control group.

**Figure 2:**
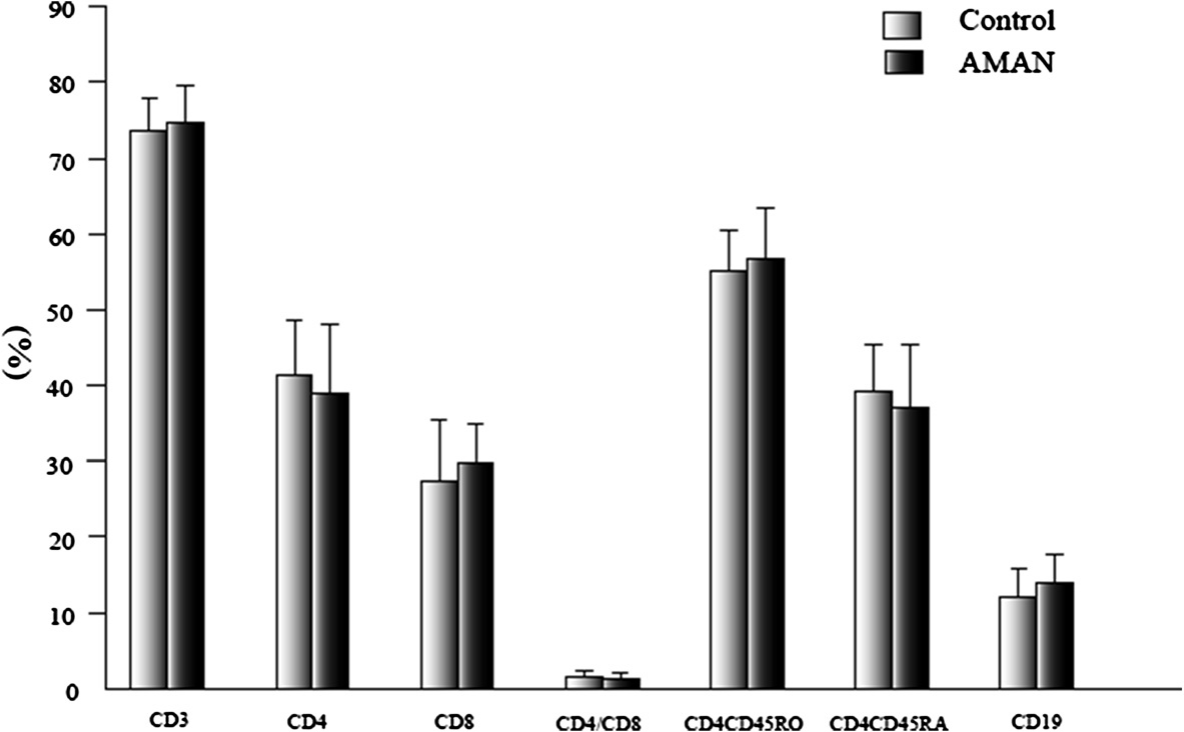
**Comparison of lymphocyte subsets in AMAN and control groups before treatment.** The data are mean ± S.D.

The percentage of CD4^+^CD45RO^+^ T cells (65.60 ± 10.41 vs 55.06 ± 5.48) was significantly higher, while the percentage of CD4^+^CD45RA^+^ T cells (29.10 ± 10.13 vs 39.24 ± 6.25) was obviously lower (*p* <0.05), in the AIDP group than in the control group, but there was no significant difference between samples drawn from the AMAN group (Figures 1 and 2).

**Figure 3:**
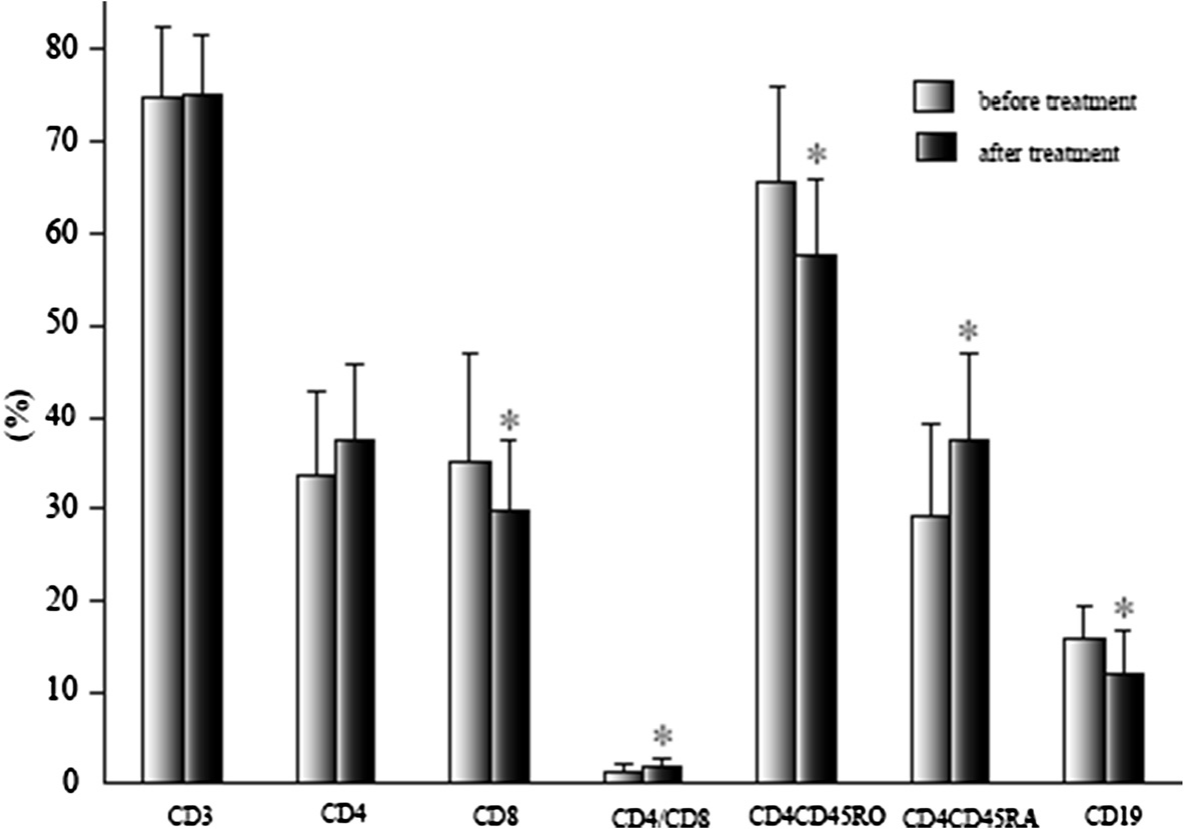
**Comparison of lymphocyte subsets in AIDP before and after treatment.** The data are mean ± S.D. *p <0.05.

**Figure 4:**
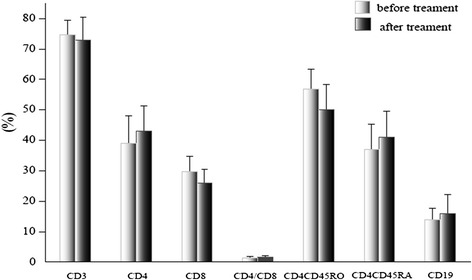
**Comparison of lymphocyte subsets in AMAN before and after treatment.** The data are mean ± S.D.

**Figure 5:**
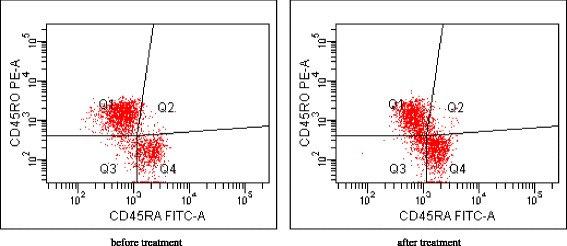
**Representative plots from individual patient from AIDP group, gated on CD4+.** The number in each quadrant represents CD45RA and CD45RO gated on CD4+.

In the AIDP group, the ratio of CD4^+^/CD8^+^ T cells (1.85 ± 1.09 vs 1.29 ± 0.80) and the percentage of CD4^+^CD45RA^+^ T cells (37.56 ± 9.22 vs 29.10 ± 10.13) increased significantly (*p* <0.05), while the percentage of CD8^+^ T (29.60 ± 7.90 vs 35.12 ± 11.94), CD4^+^CD45RO^+^ T (57.51 ± 8.45 vs 65.60 ± 10.41), and CD19^+^ B (12.11 ± 4.58 vs 15.89 ± 3.41) cells significantly decreased (*p* <0.05) after treatment. Again, there was not such a marked change following treatment in the AMAN group (Figures 3, 4, and 5).

The Hughes scores were significantly lower in both the AIDP and AMAN groups following treatment with IVIG (*p* <0.05) (Table 1).

**Table 1:** **Comparison of Hughes scores before and after treatment in each group (** $\overline{\mathbf{x}}\pm \mathbf{s}$ **)**

**Group**	**Time**	**Hughes score**
AIDP	Before treatment	3.59 ± 0.45
(n = 38)	After treatment	1.32 ± 0.67
	Falling value	2.27
	*p*	<0.05
AMAN	Before treatment	4.12 ± 0.63
(n = 26)	After treatment	2.47 ± 0.82
	Falling value	1.65
	*p*	<0.05

The change in Hughes scores was 1.95 ± 0.56 in the AIDP group and 1.73 ± 0.80 in the AMAN group, and there was no significant difference between the two groups (*p* >0.05).

## Discussion

Demyelination of motor and sensory nerves occurs in AIDP, whereas motor neurons evoke reduced amplitudes in AMAN, without demyelination. AIDP is an autoimmune disorder mediated by T and B lymphocyte systems. Pathologically, varying degrees of lymphocyte infiltration [17] and myelin sheath depigmentation can be found in AIDP, and the complement-mediated antibody attack on nerves is likely to play an important role in the pathogenesis of AIDP [18]. In AMAN, motor nerve axons, especially in Ranvier’s section, are attacked by macrophages, with varying degrees of Wallace degeneration, but rarely with inflammation and demyelination [19,20]. The etiology of GBS is not clear; however, a possible relationship with certain infections and vaccination has been documented in several studies [21-23]. GBS frequently follows a variety of presumed viral and bacterial infections [24,25], and *Campylobacter* gastroenteritis is the single most identifiable agent associated with GBS (AMAN) [26]. In subjects with GBS from northern China who developed AIDP and AMAN, the DNA-based typing of the HLA class II alleles in the two subtypes demonstrated that HLA class II epitopes are not distributed equally [27].

According to their antigen recognition receptors, T lymphocytes can be classified into two groups: TCR α/β T cells and TCR γ/δ T cells. The former make up more than 90% of T cells in peripheral blood. TCR α/β T cells, which are composed of CD3^+^CD4^+^CD8^−^ T cells and CD3^+^CD4^−^CD8^+^ T cells, play an important role in common immune response. γ/δ T cells were discovered in the past decade, and make up 0.5–10% of T cells in peripheral blood. Most γ/δ T cells express CD3^+^CD4^−^CD8^−^T, which can be activated in autoimmune diseases. T lymphocytes include helper T lymphocytes (CD4^+^T) and killer T lymphocytes (CD8^+^T). CD4^+^ T cells are heterogeneous, and include naive T cells and memory T cells, the former predominately expressing CD45RA and the latter expressing CD45RO [28,29]. In different stages of T cell development, different CD45 subtypes are expressed. Studies have shown that during the development of T cells in the thymus, a shift from CD45RO to CD45RA occurs, which marks the completion of negative selection and helps to eliminate autoreactive T cells and prevent autoimmune disease [30]. In the peripheral blood, CD4^+^CD45RA^+^ T cells can convert into CD4^+^CD45RO^+^ T cells following stimulation by antigen [30]. CD45RA and CD45RO have distinct effects on the function of B cells, and B cells can also increase the proliferation of CD45RO, in contrast to a decreased proliferation of CD45RA [31]. CD19 is an idioantigen of B lymphocytes, which also participates in their activation and signal conduction.

Our previous investigation found that if the number of CD4^+^ T or CD8^+^ T cells, or the ratio between them, changed, then immune functions may become disordered leading to a disease state in patients with GBS [5].Studies have also shown that the number of CD4^+^ T cells in patients with GBS decreased while CD8^+^ T cells increased, especially in the progressive stage. More specifically, CD4^+^CD29^+^ T cells (assist/induction of CD4^+^ T cells) increased and CD4^+^CD45RA^+^ T cells (restrain/induction of CD4^+^ T cells) decreased [6,7]. In contrast, other studies reported that the number of CD4^+^ T cells in patients with GBS increased and CD8^+^ T cells decreased [8,9]. Our previous study showed that following treatment with IVIG, CD8^+^ T and CD4^+^CD29^+^ T cells decreased in patients with GBS, whereas CD4/CD8 and CD4^+^CD45RA^+^ T cells increased [5]. To understand the changes in lymphocyte subsets in different subtypes of GBS, we carried out a further study and divided GBS patients into AIDP and AMAN groups.

In AIDP, demyelination and lymphocyte infiltration are observed. However, there is very little inflammation and demyelination in AMAN. Our research showed that the changes in lymphocyte subsets in the two GBS groups were different. In the AIDP group, the percentage of CD4^+^CD45RA^+^ T cells was markedly lower, whereas the percentage of CD4^+^CD45RO^+^ T cells was significantly higher than in the control group. The reason for this may be that CD4^+^CD45RA^+^ T cells transformed into CD4^+^CD45RO^+^ T cells after activation by antigens in the peripheral blood. This result is consistent with a study that showed that CD45RO enters into the cell cycle earlier than CD45RA stimulated by growth factors [32]. The transformation suggested that cellular immunology, especially the change in CD4^+^ T cell subsets, might play an important role in the pathogenesis of AIDP.

Cortical hormone has been the drug of choice to treat GBS. However, research has shown that routine hormone treatment cannot prevent the progression of GBS or improve prognosis [33]. At present, large doses of IVIG are the foremost immunoregulatory therapeutic method, and can ameliorate the course of GBS progression [5,34]. After therapy with IVIG, the ratio of CD4^+^/CD8^+^ T and the percentage of CD4^+^CD45RA^+^ T cells increased, while the percentage of CD8^+^ T, CD4^+^CD45RO^+^ T, and CD19^+^ B cells significantly declined in the AIDP group. We presumed that IVIG inhibited the toxic effects of CD8 killer T cells on the myelin of nerves in AIDP and by altering the distribution of CD8^+^ T, CD4^+^CD45RA^+^ T, and CD4^+^CD45RO^+^ T cells, IVIG reduced the total number of B lymphocytes. Therefore, IVIG might affect the production of autoantibodies and decrease inflammatory cell infiltration, and we found Hughes scale score was significantly lower after treatment, this result suggested IVIG could suppress peripheral nerve injury and encourage neurofunctional recovery mediated by increasing CD45RA T cell and decreasing CD45RO T cell.

In the AMAN group, changes were not significant. That is, less inflammation and demyelination were present. Although IVIG suppressed immune reactions to a certain degree and prevented aggravation of the condition, our study showed no association between the immune parameters investigated and IVIG. These findings hint that there might be other changes in immune function, and further studies are needed.

The Hughes scale score was significantly lower both in the AIDP and AMAN groups after therapy with IVIG, and the change in the score was not significantly different between the AIDP and AMAN groups. Although this study cannot explain the pathogenicity of AMAN and the mechanism by which IVIG treats it, the effect of IVIG curing AMAN is evident. Therefore, in our clinical setting, we propose administering full doses of IVIG to patients with AIDP and AMAN at the earliest stage. This might prevent aggravation of the condition, decrease paralysis of respiratory muscle, prevent tracheal incision, preclude complications, and encourage the recovery of function of damaged neurology as soon as possible.

A search of the literature found very different conclusions on lymphocyte subgroup detection in patients with GBS. We designed this study of GBS patients to make patients their own controls, before and after treatment. Such design can preclude the impact of different individuals who have suffered from infection previously. Furthermore, it can reveal the impact of disease and IVIG intervention on the immune system of an individual. GBS can be classified into two major groups: AIDP and AMAN. These groups differ in their hematological and immunological pathogenesis. Consequently, when studying lymphocyte subsets in GBS, it is important to classify GBS into AIDP or AMAN, and use patients as their own controls, before and after treatment, to minimize the effects of variation on the results.

## Conclusions

This study suggested that the changes in CD4^+^T-lymphocyte subsets might play an important role in the pathogenesis of AIDP. After treatment with IVIG, the changes in T and B-lymphocyte subsets are significant and also might play an important role in the mechanism of IVIG action against AIDP. But there were not such changes in AMAN, this study might infer that the pathogenesis and the mechanism of IVIG action against two subjects of GBS(AIDP and AMAN) are different and further studies are needed to expound these problems.
